# Excision of mesenteric lymph nodes alters gut microbiota and impairs social dominance in adult mice

**DOI:** 10.1002/brb3.3053

**Published:** 2023-05-08

**Authors:** Rui Yang, Bo‐Ya Huang, Yu‐Ning Wang, Qian Meng, Yi Guo, Shuang Wang, Xue‐Yong Yin, Hao Feng, Miao Gong, Sheng Wang, Chun‐Yu Niu, Yun Shi, Hai‐Shui Shi

**Affiliations:** ^1^ Neuroscience Research Center, Institute of Medical and Health Science Hebei Medical University Shijiazhuang China; ^2^ Hebei Key laboratory of Neurophysiology Hebei Medical University Shijiazhuang China; ^3^ Experimental Center for Teaching Hebei Medical University Shijiazhuang China; ^4^ Research Unit of Digestive Tract Microecosystem Pharmacology and Toxicology Chinese Academy of Medical Sciences Shijiazhuang China; ^5^ Department of Biochemistry and Molecular Biology Hebei Medical University Shijiazhuang China

**Keywords:** Clostridia, IL‐10, mesenteric lymph nodes, microbiota, social dominance

## Abstract

**Introduction:**

Mesenteric lymph nodes (MLNs) are central in immune anatomy. MLNs are associated with the composition of gut microbiota, affecting the central system and immune system. Gut microbiota was found to differ among individuals of different social hierarchies. Nowadays, excision of MLNs is more frequently involved in gastrointestinal surgery; however, the potential side effects of excision of MLNs on social dominance are still unknown.

**Methods:**

MLNs were removed from male mice (7–8 weeks old). Four weeks after MLN removal, social dominance test was performed to investigate social dominance; hippocampal and serum interleukin (IL)‐1β, IL‐10, and tumor necrosis factor‐alpha (TNF‐α) were investigated; and histopathology was used to evaluate local inflammation of the ileum. The composition of the gut microbiota was then examined to understand the possible mechanism, and finally intraperitoneal injection of IL‐10 was used to validate the effect of IL‐10 on social dominance.

**Results:**

There was a decrease in social dominance in the operation group compared to the control group, as well as a decrease in serum and hippocampal IL‐10 levels, but no difference in serum and hippocampal IL‐1β and TNF‐α levels, and no local inflammation of the ileum after MLN removal. 16S rRNA sequencing analysis showed that the relative abundance of the class Clostridia was decreased in the operation group. This decrease was positively associated with serum IL‐10 levels. Furthermore, intraperitoneal injection of IL‐10 in a subset of mice increased social dominance.

**Conclusions:**

Our findings suggested that MLNs contributed to maintaining social dominance, which might be associated with reduced IL‐10 and the imbalance of specific flora in gut microbiota.

## Graphical abstract

By analyzing the alteration of gut microbiota, immune system, and social dominance after resection of mesenteric lymph nodes, we found that mesenteric lymph node resection may lead to a decrease in social dominance by altering gut microbiota and immune system.

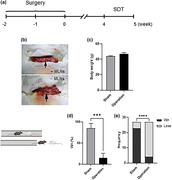

## 1 INTRODUCTION

Mesenteric lymph nodes (MLNs) are central in immune anatomy and the central checkpoint for mucosal immunity and other immunizations (Macpherson & Smith, 2006). MLNs, the most prominent lymph nodes in the body (Murphy & Weaver, 2016), are located in the connective tissue that tethers the intestine to the rear wall of the abdomen. Gut microbiota can be cultured in the spleen after excision of MLNs (Macpherson & Uhr, 2004). They drain lymph from the small intestine and play a crucial role in shaping responses to intestinal antigens. Recent studies have reported MLNs are important for inducting tolerance toward intestinal commensal (Lyu et al., 2022; Pezoldt et al., 2018), and diversification of gut microbiota relies on the regulatory T cell (Treg) induced by MLNs (Kawamoto et al., 2014). Meanwhile, Treg promotes the production of interleukin (IL) 10—an anti‐inflammatory cytokine—which is essential to reduce inflammation and protect the functions of the central nervous system (CNS) (Böhm et al., 2015; Proto et al., 2018). IL‐10 deficit is related to an imbalance in excitatory and inhibitory (E/I) transmission in the medial prefrontal cortex (Yang, Liu, et al., 2021), and an imbalance in E/I impairs social dominance (Tan et al., 2018).

Social hierarchy determines health and survival in humans and other animals (Marmot et al., 1978; Snyder‐Mackler et al., 2020). Synaptic efficacy in medial prefrontal cortex‐modulated social dominance (Wang et al., 2011) is well known. Additionally, Kumaran's study has found that the activity of hippocampal neurons was correlated with social dominance (Kumaran et al., 2012). Social dominance is associated with many brain regions, such as the amygdala, striatum, intraparietal sulcus, medial prefrontal cortex, and hippocampus (Watanabe & Yamamoto, 2015). The role of the hippocampus in social dominance is also beginning to receive attention. The hippocampus interacts with the amygdala to influence social dominance through social dominance‐related interaction and social dominance learning (Kumaran et al., 2016; Li et al., 2021). Meanwhile, M2‐like microglia in the hippocampus have also been found to be associated with social hierarchy (Piirainen et al., 2021) and suggest the importance of the hippocampus in social dominance. However, more research is needed to elucidate how the hippocampus regulates social dominance. Furthermore, gut microbiota was found to differ among individuals of different social dominance (Yun et al., 2018; Zeng et al., 2022), but how gut microbiota influence social dominance remains unclear.

Cells of MLNs play an important role in maintaining the stability of intestinal flora (Lyu et al., 2022), and gut microbiota impacts neurophysiology and modulates social behavior (Vuong et al., 2017). Multiple pathways link the gut to CNS, such as immune, metabolic, circulatory, and neuronal pathways (Cryan & Dinan, 2012; Schroeder & Bäckhed, 2016). Among them, the microbial byproduct short‐chain fatty acids (SCFAs) can regulate host behavior and SCFA (Sharon et al., 2016; Van De Wouw et al., 2018), and SCFA can enhance the mTOR pathway to increase the synthesis of IL‐10 (Luu et al., 2019). Based on this, a disturbing brain–gut axis might contribute to social dominance by impairing the synthesis of IL‐10.

The present study aimed to explore the regulatory effect of MLNs on social dominance and determine whether the immune system and gut microbiota are involved in this regulation by removing MLNs. We found that excision of MLNs decreased social dominance in adult mice, altered the composition of gut microbiota, and reduced IL‐10 in serum and hippocampus, respectively. These results suggest a close relationship between MLNs and immune balance and the stability of intestinal microflora microenvironment, which provides an experimental basis further to investigate the role of MLNs in social dominance.

## 2 MATERIALS AND METHODS

### 2.1 Animals

Adult Institute of Cancer Research (ICR) male mice (7–8 weeks old on arrival) were obtained from Beijing Vital River Laboratory Animal Technology Co., Ltd. Mice were housed in a controlled environment with a temperature of 22 ± 2°C, humidity of 50% ± 5%, a 12‐h light/dark (lights on at 8:00 p.m. and off at 8:00 a.m.) cycle, and free access to food and water. The operation group comprised the mice that received MLN dissection, while the mice that received sham surgery were in the sham group. All animal experiments were approved by the Local Committee on Animal Care and Use and Protection of the Hebei Medical University and followed the National Institutes of Health Guide for the Care and Use of Laboratory Animals guidelines.

### 2.2 Experimental design

#### 2.2.1 Experiment 1

First, the mice were acclimated to the environment, and then underwent MLN dissection (see Section 2.2 for detailed methods), which was recorded as week −2 to week 0. After the surgery, the mice were given 4 weeks to recover and establish a stable social hierarchy, including the time for training before the social dominance test (SDT). The SDT (see Section 2.3 for detailed methods) was conducted in week 5. The mice were randomly assigned to two different groups using a random number table, and four or five same‐sex mice were kept in one cage, with two cages per group.

#### 2.2.2 Experiment 2

Mice were given 1 week to acclimate to the environment, referred to as week −1 to week 0. Subsequently, pretraining for SDT was conducted in week 1, and mice were allowed to establish a stable social hierarchy from week −1 to week 1. Testing was conducted in week 2, and IL‐10 injections were administered for five consecutive days after testing (see Section 2.8 for detailed methods). SDT was conducted after the injections are administered. The mice were randomly assigned to two different groups using a random number table, and four same‐sex mice were kept in one cage, with five cages per group.

### 2.3 Intestinal surgery

The procedure of the excision of MLNs was based on a previous study (Worbs et al., 2006). In brief, under anesthesia with pentobarbital, excision of MLNs was performed by microdissection along the length of the superior mesenteric artery to the aortic root. The exposed bowel was moistened with warm saline gauze. Finally, the abdomen was sutured after confirming the viability of the bowel. In the sham group, the above operation was completed except for removing MLNs.

### 2.4 Social dominance test

The SDT was conducted based on prior research (Fan et al., 2019; Hao et al., 2022; Huang et al., 2022). A clear plexiglass cylindrical tube (length, 60 cm; diameter, 4 cm) was used to measure the social dominance of the mice. The mice were required to adapt to the tube before the experiment. Two test mice were placed at the entrance of two sides of the tube, and the mice were allowed to enter the tube at the same time, until one mouse was pushed out of the tube; the mouse that was pushed out of the tube was recorded as a failure. The same group of nine mice was divided into nine ranks (experiment 2: four mice were divided into four ranks in the same cage) according to the total number of wins (if the number of wins was the same, the two mice were ranked according to the relationship between wins and losses). First, social dominance was compared within the same group and the mice were ranked accordingly. Then, mice were used from different groups but with the same ranking of social dominance for between‐group comparisons. Intragroup tests were performed three times. Comparisons between groups were made according to the ranking of mice in the same group; after three tests, the social ranking was compared according to the number of wins. The cylinder was cleaned with 75% alcohol between each comparison (including intragroup and intergroup comparisons).

### 2.5 Isolation of mouse hippocampus samples

In brief, after euthanizing the mouse, the scalp was incised and the skull was carefully opened, referring to previous research (Jaszczyk et al., 2022). Since the hippocampal tissue was located at the bottom of the cerebral cortex, it was delicately separated from the cerebral cortex and collected for further use.

### 2.6 Histopathology

The ileum was collected and fixed in 10% paraformaldehyde. After routine processing including paraffin embedding, tissue sectioning, and slide mounting, tissues were stained with hematoxylin and eosin (HE). Images were obtained with a stereoscopic optical microscope.

### 2.7 Enzyme‐linked immunosorbent assay

At the end of 5 weeks, blood samples of mice were collected under anesthesia with 2% pentobarbital (0.2 mL). The mice were euthanized with an overdose of pentobarbital. Blood was immediately centrifuged at 4000 rpm for 10 min at 4°C. The upper serum was taken and stored at −80°C until analysis. Hippocampus was frozen in liquid nitrogen and stored at −80°C until analysis. The content of IL‐1β (Mlbio cat. no. m1063132), IL‐10 (Mlbio cat. no. m1002285), and TNF‐α (Mlbio cat. no. m1002095) in serum was measured by enzyme‐linked immunosorbent assay.

### 2.8 Microbial community composition analysis

Samples of intestinal contents from the ileum were collected and frozen at −80°C. The total DNA of the samples was extracted (*n* = 5) using QIAamp Fast DNA Stool Mini Kit (QIAGEN, Germany). The DNA concentration and purity were determined by a NanoDrop 2000 UV‐vis spectrophotometer (Thermo Scientific, Wilmington, USA), and the DNA quality was checked by 1% agarose gel electrophoresis. PCR amplification was performed with the primers targeting 11 V3–V4 regions: 338F (5′‐ACTCCTACGGGAGGCAGCA‐3′) and 806R (5′‐ GGACTACNNGGGTNTCTAAT‐3′). The sequencing was then performed by the OE Biotechnology company in Shanghai using the Illumina Miseq system. The raw data were denoised by DADA noise reduction of the QIIMA2 platform to obtain the ASV representative sequences. The α‐diversity and β‐diversity were analyzed using the QIIME software package (version 1.8.0).

### 2.9 IL‐10 treatment

IL‐10 treatment is performed as described in previous work (Han et al., 2015). Dominance rank 3 was assigned to recombinant mouse IL‐10 protein (ABclonal. RP01465) or saline. For 5 days, mice were treated by intraperitoneal injection of saline or a combination of cytokines (100 ng of IL‐10 and 100 ng of IL‐4), and social dominance was assessed after 24 h postinjection.

### 2.10 Statistical analysis

Data were expressed as the mean ± SEM. Unpaired two‐tailed *t*‐test and Mann–Whitney test were used for data analysis. All calculations were performed using GraphPad Prism 9.0. Based on Bray–Curtis distances, principal coordinates analysis (PCoA) was conducted to compare changes in microbiota communities between samples. Values of *p* < .05 were considered statistically significant.

## 3 RESULTS

### 3.1 Excision of MLNs degraded social dominance

**FIGURE 1:**
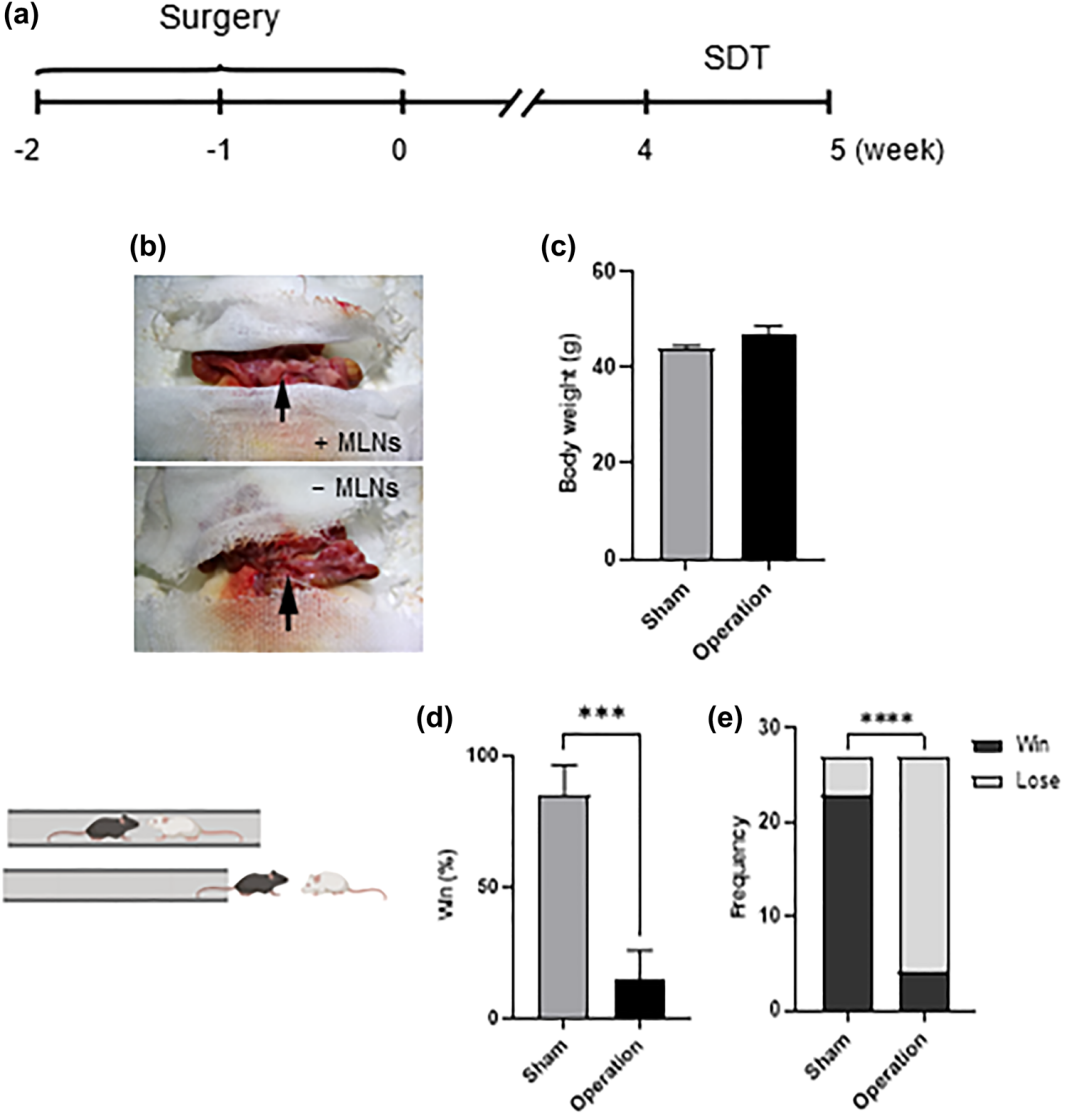
Excision of mesenteric lymph nodes (MLNs) degraded social dominance. (A) Experimental procedure. (B) Mice undergoing excision of MLNs (operation) and sham operated mice (*n* = 9 per group). (C) Body weight measured (*n* = 9 per group). Social dominance test (SDT) was conducted in week 4. In the SDT, winning percentage and number of wins in sham and operation groups were shown in panels D and E, respectively (*n* = 9 per group). The data are expressed as mean ± SEM. ***p < .001; ****p < .0001.

The experimental procedure was shown in Figure 1A. To test the effects of excision of MLNs, we surgically removed the MLNs in adult mice (Figure 1B). Body weight was measured 1 day before SDT, and the results showed that body weight had no significant difference between the two groups (*p* = .076, Figure 1C). To determine whether excision of MLNs induced social dominance alteration, the SDT was performed to investigate social dominance in adult mice. Compared with the sham group, excision of MLNs decreased social dominance (*p* = .000, Figure 1D and *p* = .000, Figure 1E). Thus, excision of MLNs degraded social dominance in adult mice.

### 3.2 Excision of MLNs reduced the levels of IL‐10 in both serum and hippocampus

**FIGURE 2:**
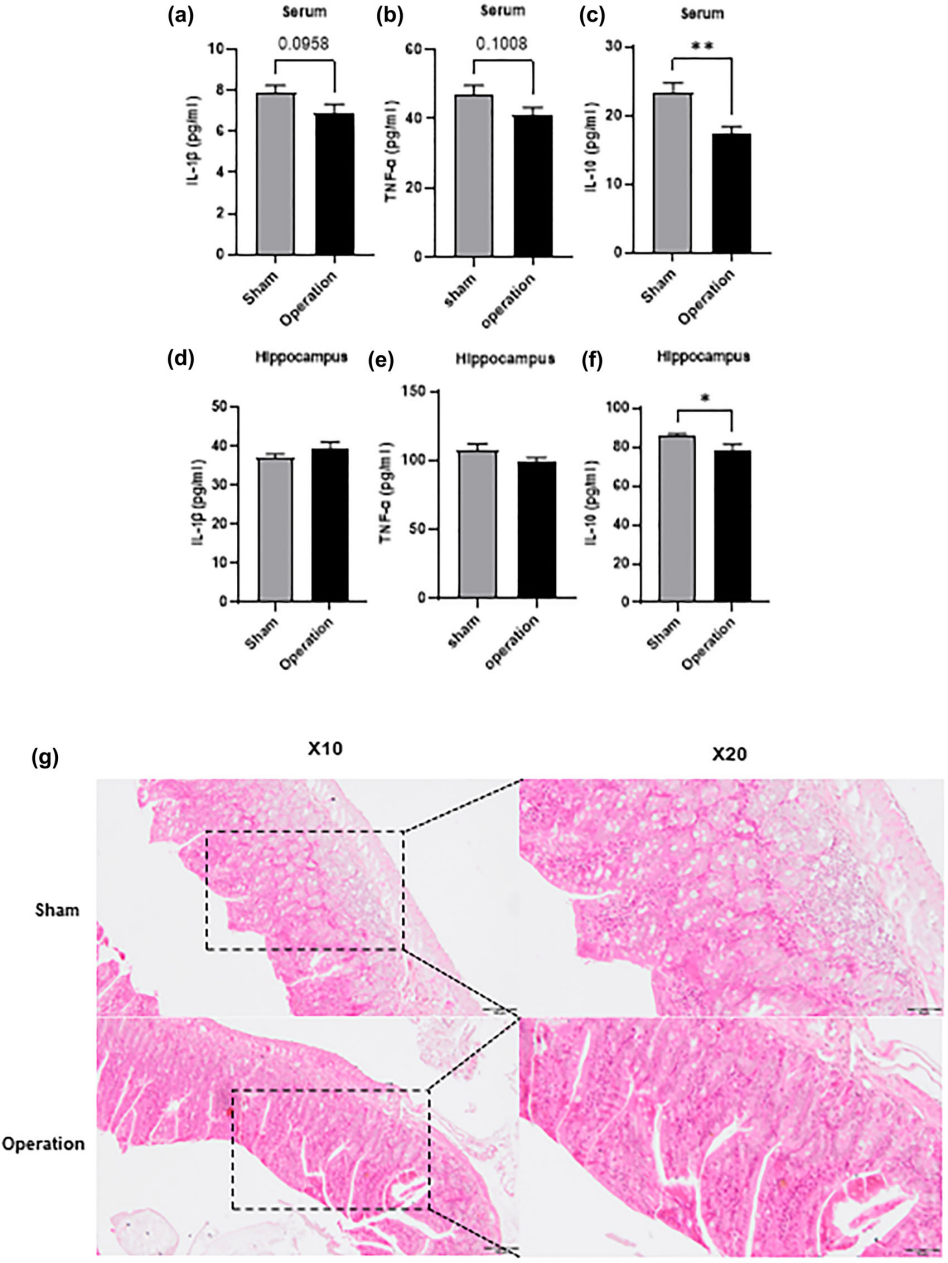
Excision of mesenteric lymph nodes (MLNs) reduced the levels of IL‐10 in both serum and hippocampus. (A) Levels of IL‐1β in serum (*n* = 9 per group). (B) Levels of TNF‐α in serum (*n* = 9 per group). (C) Levels of IL‐10 in serum (*n* = 9 per group). (D) Levels of IL‐1β in hippocampus (*n* = 7 per group). (E) Levels of TNF‐α in hippocampus (*n* = 7 per group). (F) Levels of IL‐10 in hippocampus (*n* = 7 per group). (G) HE staining of ileum tissue from sham and operation groups, taken with 10× and 20× magnifications. Scale bars, 100 and 50 μm. *n* = 4 for each group. The data are expressed as mean ± SEM. *p < .05; **p < .01.

Recent research reported that MLNs play a crucial role in the periphery immune system (Pasztoi et al., 2017). To confirm the effect of excision of MLNs on the immune system, we first measured the levels of IL‐1β, TNF‐α, and IL‐10 in serum and found that there were no significant changes in the levels of IL‐1β (*p* = .096, Figure 2A) and TNF‐α (*p* = .101, Figure 2B); however, there was a significant decrease in the levels of IL‐10 dominance after excision.

Alteration of the periphery immune system could affect CNS immune system and induce anxiety‐like behavior (Hanscom et al., 2021; Wang et al., 2018), therefore we further measured the levels of IL‐1β, TNF‐α, and IL‐10 in the hippocampus. Consistent with the data in serum, excision of MLNs showed no effect on the levels of IL‐1β (*p* = .309, Figure 2D) and TNF‐α (*p* = .156, Figure 2E), but decreased the level of IL‐10 (*p* = .041, Figure 2F) in the hippocampus.

MLNs are central in mucosal immunity (Macpherson & Smith, 2006) and excision of MLNs may lead to a local immune response in the intestine. Therefore, HE staining was performed to determine whether excision of MLNs affected the mucosal immunity of the intestine. Our results showed that there was no ulceration, inflammation, or edema in the ileum (Figure 2G), indicating that excision of MLNs did not affect mucosal immunity in situ.

The above results suggest that excision of MLNs did not trigger immune responses in the mucosa, peripheral circulation, and CNS, but it reduced the levels of IL‐10 in serum and hippocampus. These results suggested that excision of MLNs did not cause local and systemic inflammatory responses. However, it indeed reduced the levels of IL‐10 in both serum and hippocampus, which may be an important cause of degraded social dominance.

### 3.3 Excision of MLNs altered the composition of gut microbiota in adult mice

**FIGURE 3:**
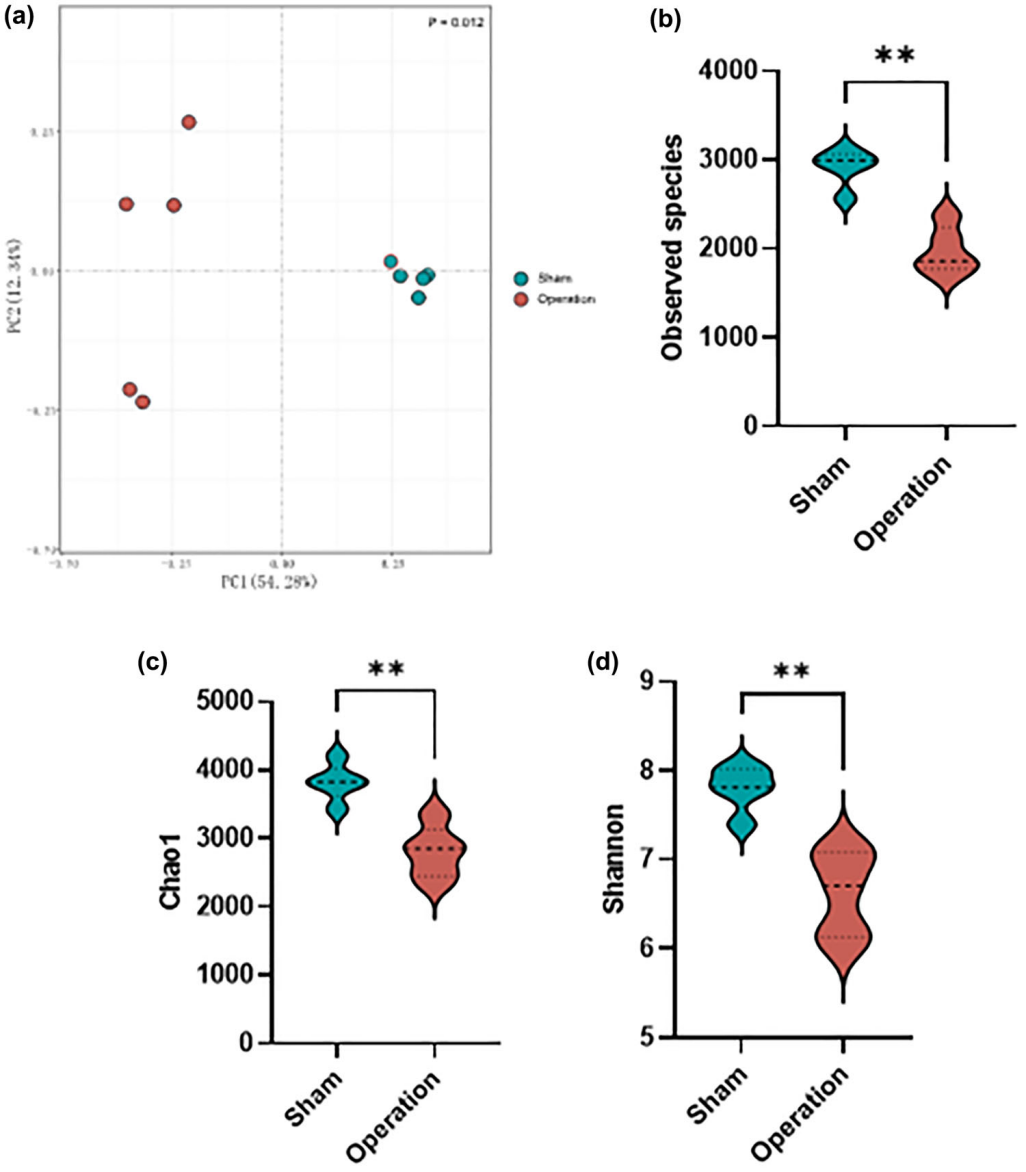
Excision of mesenteric lymph nodes (MLNs) altered the composition of gut microbiota in adult mice. (A) Principal coordinates analysis (PCoA) plot (based on Bray–Curtis distances). (B) The observed species index, (C) the Chao1 estimator, and (D) the Shannon index. *n* = 5 per group. **p < .01.

IL‐10 can maintain the homeostasis of gut microbiota (Neumann et al., 2019). Based on the facts that excision of MLNs reduced the levels of IL‐10 and that MLNs were involved in maintaining peripheral tolerance toward commensal antigens (Pezoldt et al., 2018), we assumed that excision of MLNs was likely to have an impact on the composition or abundance of gut microbiota. To prove it, we performed 16S rRNA gene sequencing analysis to detect α‐diversity, β‐diversity, and composition of gut microbiota. PCoA of the UniFrac distance for β‐diversity revealed a clear separation among groups; there were significantly different communities in the ileum contents of the operation group and the sham group (*p* = 0.012, Figure 3A). The observed species index (*p* = .008, Figure 3B) and Chao1 estimator decreased in the operation group (*p* = .008, Figure 3C). Similarly, the Shannon index also decreased in the operation group (*p* = .008, Figure 3D). These results suggest that the diversity and overall abundance of gut microbiota were widely reduced after excision of MLNs, which implied the disruption of gut microbiota homeostasis.

### 3.4 Reduction in class *Clostridia* is associated with reduction of IL‐10 in serum

**FIGURE 4:**
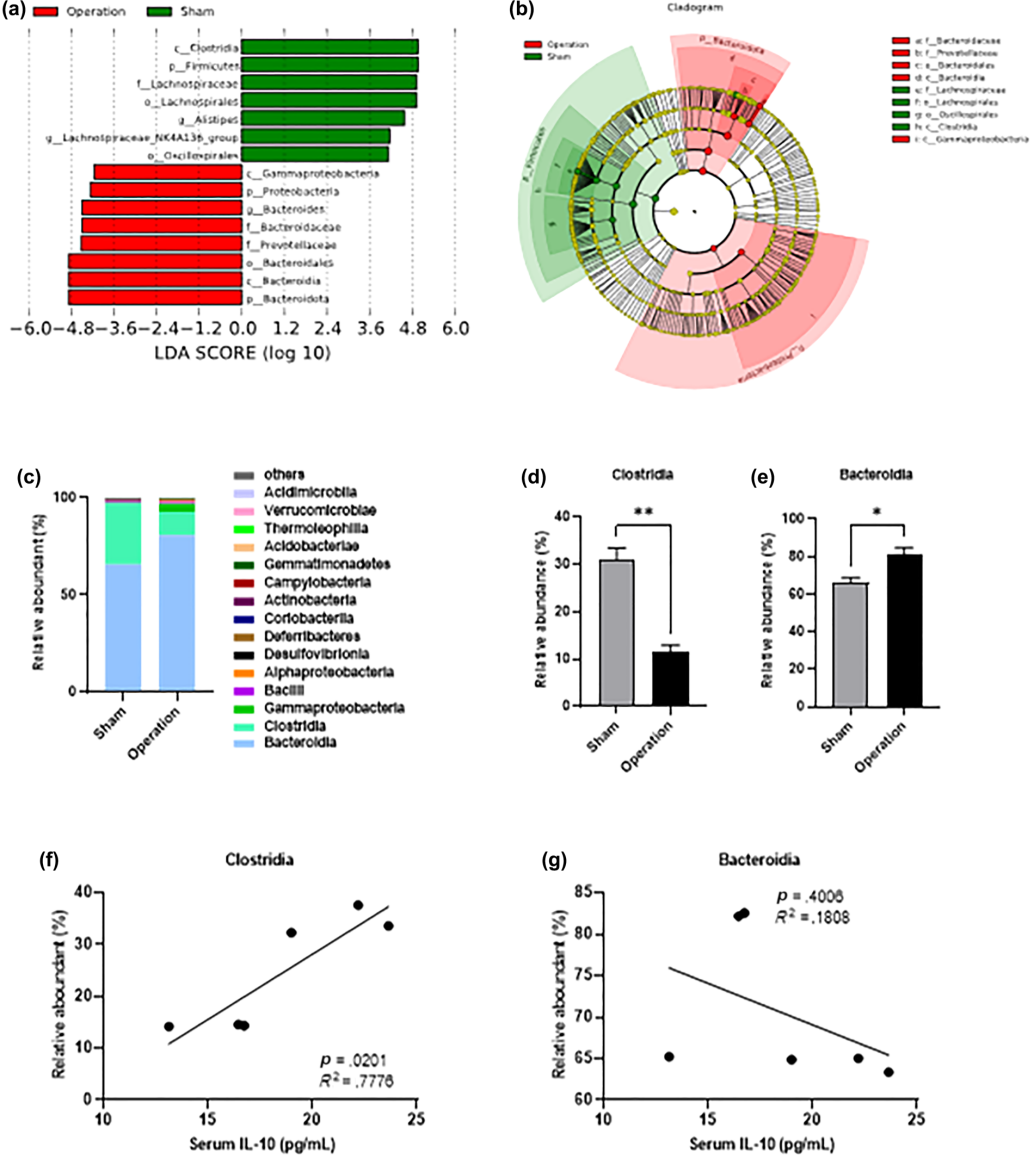
Reduction in class *Clostridia* is associated with reduction of IL‐10 in serum. (A) Linear discriminant analysis (LDA) coupled with effect size measurements (LEfSe) showed the composition differences of gut microbiota between the sham group and the operation group. (B) Cladogram of species annotated (LEfSe: *p* < .05, *q* < .1, LDA SCORE > 4.0). (C) The composition of gut microbiota at class level. (D) Comparison of the relative abundance of *Clostridia* (D) and *Bacteroidia* (E). (F) Correlation analysis between *Bacteroidia* and serum IL‐10. (G) Correlation analysis between *Bacteroidia* and serum IL‐10. *n* = 5 per group. *p < .05; **p < .01; ***p < .001; ****p < .0001.

By linear discriminant analysis coupled with effect size measurements (LEfSe), we assessed the composition differences of gut microbiota between the sham group and the operation group (Figure 4A,B). There were seven microbial groups (*Firmicutes*, *Clostridia*, *Lachnospirales*, *Oscillospirales*, *Lachnospiraceae*, *Alistipes*, and *Lachnospiraceae_NK4A136_group*) with significant differences in the sham group, and eight microbial groups (Bacteroidota, Proteobacteria, Bacteroidia, Gammaproteobacteria, Bacteroidales, Prevotellaceae, Bacteroidaceae, and Bacteroides) with significant differences in the operation group. Analysis of these microbial groups in different groups (Figure 4A) revealed that most of the microbial groups belong to *Clostridia* in the sham group and most of the microbial groups belong to *Bacteroidia* in the operation group. Combined with the results from LEfSe and Cladogram, the sham group was characterized by *Clostridia*, and the operation group was characterized by *Bacteroidia*. *Bacteroidia* and *Clostridia* were the two most abundant at the class level (Figure 4C). According to LEfSe results, we analyzed the changes in the relative abundance of microbiota. After excision of MLNs, *Clostridia* was decreased (*p* = .008, Figure 4D) and *Bacteroidia* was increased (*p* = .016, Figure 4E). These results suggest excision of MLNs induced the reduction of *Clostridia* and its members, as well as the increase of *Bacteroidia* and its members. IL‐10 is required for maintaining homeostasis of the gut microbiota (Neumann et al., 2019), therefore we analyzed the relationship between *Clostridia*, *Bacteroidia*, and serum IL‐10. The results showed that *Clostridia* was positively correlated with serum IL‐10 (*p* = .020, Figure 4F); however, there was no correlation between *Bacteroidia* and serum IL‐10 (*p* = .401, Figure 4G).

These results indicate that excision of MLNs disrupted the homeostasis of gut microbiota mainly by changing the abundance and composition of *Clostridia* and *Bacteroidia*, and the change of *Clostridia* is closely related to the downregulation of IL‐10.

### 3.5 Supplement of IL‐10 promoted social dominance

**FIGURE 5:**
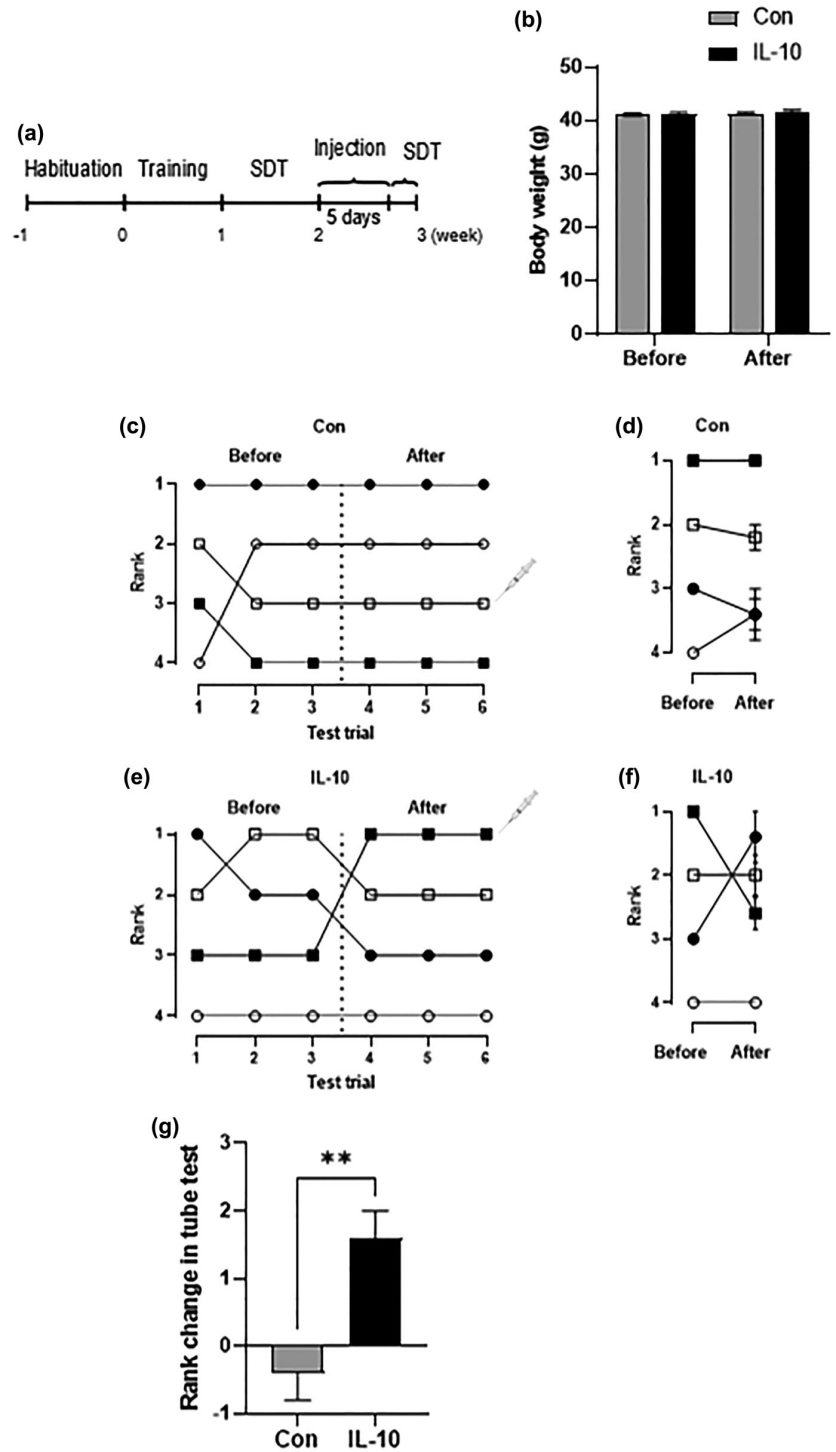
Supplement of IL‐10 promoted social dominance. (A) Experimental procedure. (B) Body weight change before and after IL‐10 treatment. (C) Example of the rank positions of one cage of mice in the control group. (D) Summary graph for five cages measured in control group. (E) Example of the rank positions of one cage of mice in IL‐10 group. (F) Summary graph for five cages measured in IL‐10 group. (G) Changes in social dominance after injections. *n* = 5 per group. **p < .01.

To investigate whether IL‐10 could impact social dominance, we treated dominance rank 3 mice with IL‐10. The experimental schedule is described in Figure 5A. Body weight had no difference between the control group and the IL‐10 group before and after IL‐10 treatment (Figure 5B). Then, SDT was performed to assess the change in social dominance. Social dominance remained stable after the injection of saline (Figure 5C,D), but social dominance increased after IL‐10 treatment (Figure 5E,F). Compared with the control group, IL‐10 treatment promoted social dominance (*p* = .008, Figure 5G).

## 4 DISCUSSION

In the present study, we elucidated the side effect of excision of MLNs. We found that excision of MLNs degraded social dominance and had an influence on the immune system and gut microbiota. In addition, we found that excision of MLNs decreased levels of IL‐10 in both serum and hippocampus. Meanwhile, significant alterations in gut microbiota composition, abundance, and function were observed after excision of MLNs. These findings are the first demonstration that excision of MLNs alters gut microbiota and impairs social dominance in adult mice.

The present results showed that there was no elevation of local inflammation in the ileum, as well as systemic pro‐inflammation factors; however, the levels of IL‐10 were reduced in the hippocampus and serum after excision of MLNs. MLNs primarily drain lymph from the small intestine (Murphy & Weaver, 2016), and although the small intestine has relatively fewer flora and immune cells compared to the large intestine, it also has its unique gut microbiota, which can affect the host (Belkaid & Hand, 2014; Kastl et al., 2020). The reason for selecting the ileum is that it contains a large amount of immune cells and can generate immune responses to intestinal antigens when compared to other parts of the small intestine (Murphy & Weaver, 2016). As a result, MLNs resection has a more significant effect on the ileum. We believe there are two reasons why there was no increase in local inflammation or systemic pro‐inflammatory factors. First, in addition to MLNs, the intestine contains a large amount of lymphoid tissue, including lymphoid aggregates and isolated lymphoid follicles (Murphy & Weaver, 2016). The remaining lymphoid tissue may compensate for some of the loss of lymph nodes. Second, the immune system has remarkable plasticity and can adapt to changes in lymphoid tissue (Mackay & Kallies, 2017; Streitz et al., 2013). We believe that these may be the reasons why the intestinal mucosal barrier remains healthy and pro‐inflammatory factors do not increase. MLNs have a special microenvironment that promotes the synthesis of Tregs (Benson et al., 2007; Coombes et al., 2007; Hammerschmidt et al., 2008; Pezoldt et al., 2018), which cannot be compensated for by other lymph nodes. This is why there are differences in IL‐10 and other cytokine changes. First, the proportion of Treg is higher in MLNs than in other peripheral lymph nodes (Siewert et al., 2007; Sun et al., 2007), since MLNs provide a microenvironment for the de novo generation of Treg (Pezoldt et al., 2018). Retinal dehydrogenase also had a high expression in MLNs (Coombes et al., 2007; Hammerschmidt et al., 2008) and promoted the production of retinoic acid, which induces the differentiation of Treg (Benson et al., 2007; Coombes et al., 2007). Importantly, the function of Treg is dependent on the secretion of IL‐10 (O'garra et al., 2008; Pasztoi et al., 2017). Thereby, excision of MLNs might disrupt the generation of Treg and further reduce the levels of serum IL‐10. Second, the reduction of *Clostridia* might involve the reduction of IL‐10 in serum. Under *Clostridia*, *Lachnospiraceae_NK4A136_group* and *Lachnospiraceae* are reported to be positively associated with the expression of IL‐10 (Chen et al., 2021; Sorbara et al., 2020). Meanwhile, butyrate, a metabolite of *Lachnospiraceae* (Sorbara et al., 2020), can promote the expression of IL‐10 (Paparo et al., 2021). We also found that excision of MLNs decreased *Alistipes*, a microbial that has been reported to increase in IL‐10‐increased mice (Wan et al., 2021). In the study, *Clostridia* significantly reduced after excision of MLNs, and serum IL‐10 was positively associated with the abundance of *Clostridia*. Therefore, the reduction of *Clostridia* may lead to the reduction of IL‐10 in serum.

Cytokines, such as IL‐10, may cross the brain–blood barrier and entry to CNS (Banks et al., 1995). The decrease of serum IL‐10 might reduce the transport of IL‐10 from blood to CNS, which resulted in the reduction of IL‐10 in the hippocampus. IL‐10, as an anti‐inflammation cytokine, has been reported to be able to change the function of CNS (Nakata et al., 2016). The present study showed that social dominance was degraded after excision of MLNs, combined with the reduction of IL‐10 in the hippocampus. Piirainen et al. (2021) reduced M2‐like polarization in hippocampal microglia by knocking down CX3CR1 and demonstrated that M2‐like polarization in microglia is positively correlated with social dominance. IL‐10 has similar anti‐inflammatory effects as CX3CR1 in microglia and IL‐10 also promotes microglia cells toward M2‐like polarization (Han et al., 2015; Ishida et al., 2017), therefore the reduction of hippocampal IL‐10 may lead to a reduction in M2‐like polarization of microglia through CX3CR1 knockout as well, resulting in a decrease in social dominance. This result is also consistent with previous studies in which animals with low social dominance exhibited a higher pro‐inflammatory response (Lee et al., 2022; Snyder‐Mackler et al., 2016).

MLNs maintain peripheral tolerance toward commensal antigens (Lyu et al., 2022; Pezoldt et al., 2018). Gut microbiota imprint tolerogenic properties into MLNs stromal cells, and these stromal cells maintain tolerance to gut microbiota by interacting with resident dendritic cells (Pezoldt et al., 2018). In the present study, we found that the diversity and richness of bacteria declined after excision of MLNs, we considered that excision of MLNs disrupted tolerance toward microbiota and induced alteration of microbiota. From existing research, a possible mechanism is that IgA deficiency can lead to changes in the composition of gut microbiota. MLNs are the main source of IgA (Mcwilliams et al., 1977). IgA protects the symbiotic bacteria and inhibits the growth of pathogenic bacteria (Fadlallah et al., 2018), therefore reduction of IgA in the gut can negatively affect the composition of the microbiota (Donaldson et al., 2018; Fadlallah et al., 2018; Kubinak et al., 2015; Rigoni et al., 2016). Based on this view, MLNs can be seen as the center of maintaining the stability of gut microbiota. The predominant bacterium in the control group was *Clostridia*, which was closely associated with IL‐10 as previously described. Additionally, there are four different microbial groups (*Bacteroidales*, *Bacteroidaceae*, *Prevotellaceae*, and *Bacteroides*) belonging to *Bacteroidia* in the operation group, of which *Bacteroidales* is associated with inflammation (Xu et al., 2020), but there is no evidence that *Bacteroidales* is related to IL‐10. Previous studies have shown that *Bacteroidaceae* (Yang, Ye, et al., 2021), *Prevotellaceae* (Qu et al., 2021; Yu et al., 2021), and *Bacteroides* (Wan et al., 2021) were reduced in mice with the elevation of IL‐10. Similarly, in the present study, the decreased IL‐10 was accompanied by an increase in the abundance of *Bacteroidaceae*, *Prevotellaceae*, and *Bacteroides* after excision of MLNs. Despite all this, there is no correlation between *Bacteroidia* and serum IL‐10. Overall, we considered that *Clostridia* predominates in the reduction of serum IL‐10, not *Bacteroidia*.

In conclusion, our study demonstrated that excision of MLNs disrupted the immune system, altered gut microbiota, and degraded social dominance in adult mice. The mechanisms underlying the effects of excision of MLNs on social dominance warrant further investigation, and it is necessary to confirm whether the effects are also applicable to the clinical population. In conclusion, our findings extend our understanding of the complications after excision of MLNs, emphasize the importance of social dominance, and provide directions for future research and clinical practice.

## AUTHOR CONTRIBUTIONS

**Rui Yang**: Investigation; data curation; methodology; validation; formal analysis; writing—original draft. **Bo‐Ya Huang**: Investigation; data curation; methodology. **Yu‐Ning Wang**: Investigation; data curation. **Qian Meng**: Investigation. **Yi Guo**: Investigation. **Shuang Wang**: Investigation. **Xue‐Yong Yin**: Investigation. **Hao Feng**: Investigation. **Miao Gong**: Methodology; investigation. **Sheng Wang**: Investigation; data curation. **Chun‐Yu Niu**: Supervision. **Yun Shi**: Validation; methodology. **Haishui Shi**: Conceptualization; writing—review and editing; supervision; project administration.

## CONFLICT OF INTEREST STATEMENT

The authors declare no conflicts of interest.

### PEER REVIEW

The peer review history for this article is available at https://publons.com/publon/10.1002/brb3.3053.

## Data Availability

The data that support the findings of this study are available from the corresponding author upon reasonable request.
